# The effects of physical therapeutic agents on serum levels of stress hormones in patients with osteoarthritis

**DOI:** 10.1097/MD.0000000000004660

**Published:** 2016-09-02

**Authors:** Şükrü Burak Tönük, Erdinc Serin, Fikriye Figen Ayhan, Zeynep Rezan Yorgancioglu

**Affiliations:** a Department of Physical Medicine and Rehabilitation, School of Medicine, Abant Izzet Baysal University, Bolu; b Department of Medical Biochemistry, Istanbul Research and Education Hospital, Istanbul; c Department of Physical Medicine and Rehabilitation, Ankara Research and Education Hospital, Ankara, Turkey.

**Keywords:** osteoarthritis, pain, stress hormones, therapeutic ultrasound, thermotherapy, transcutaneous electrical nerve stimulation

## Abstract

To investigate the effects of physical agents on the levels of stress hormones in patients with osteoarthritis (OA).

Transcutaneous electrical nerve stimulation, hot packs, and therapeutic ultrasound were applied to the lumbar region and knees of patients with OA. Blood samples were taken for the measurement of the serum levels of glucose, insulin (INS), growth hormone (GH), prolactin (PRL), cortisol (COR), and plasma adrenocorticotropic hormone (ACTH) immediately before and after the 1st session, to investigate the acute effects of those physical agents on the endocrine system. The hormone levels were also measured every 5 sessions in a total of 10 sessions. The treatment response was also evaluated by using the visual analogue scale (VAS), Roland Morris Disability Questionnaire (RMDQ), and Western Ontario and McMaster Universities Arthritis Index (WOMAC) throughout the therapy period.

After the 1st session, there was a decrease in INS levels and a mild decrease in PRL levels (*P* = 0.001 and *P* < 0.05, respectively). Throughout the 10-session therapy period, the INS levels increased, whereas the ACTH and COR levels decreased (*P* < 0.05 for all). The VAS-spine, RMDQ, VAS-knee, and WOMAC scores decreased (*P* = 0.001 for VAS-spine and *P* < 0.001 for all others). A positive correlation was detected between the changes in serum COR and WOMAC-pain score (*P* < 0.05).

Although the combination therapy caused changes in INS level accompanied with steady glucose levels, the application of physical agents did not adversely affect the hormone levels. The decrease in ACTH and COR levels may be attributed to the analgesic effect of agents and may be an indicator of patient comfort through a central action.

## 1 Introduction

Physical agents have been safely used for many years as a treatment method for musculoskeletal disorders. In clinical practice, physical agents are commonly used as a combination of electrotherapy and thermotherapy techniques. Thermotherapy is useful as it reduces pain, muscle spasm, and joint stiffness, while also enhancing local blood flow, local metabolism, and nerve transduction speed. Thermotherapy increases musculoskeletal tissue regeneration, alleviates pain, and increases the range of motion by reducing joint stiffness. Its adverse effects include fluid and electrolyte loss, low blood pressure, fainting, and burns. Hot packs, whirlpool, paraffin, and infrared lamps are frequently used as superficial heating agents, whereas ultrasound (US) and microwave diathermy are used as deep heating agents.

Therapeutic US is the most commonly used deep heating agent in clinical practice. Its biophysical effects are classified according to its thermal and nonthermal properties. The thermal effects of therapeutic US include increased metabolic speed, control of pain and muscle spasm, increased nerve conduction speed, and enhanced circulation and soft tissue extensibility. These thermal effects depend on the application time, power, and frequency values. The mechanical (nonthermal) effects of US include the cavitation effect, microsteaming, and acoustic steaming. US has a potential chondroprotective effect on the osteoarthritic cartilage and is one of the safest treatment options for knee osteoarthritis (OA) and chronic low back pain.^[1–3]^

Many clinicians apply US in conjunction with electrotherapy. Transcutaneous electrical nerve stimulation (TENS) is one of the most commonly performed electrotherapy techniques. The electrical current produced by the TENS device alleviates pain by stimulating the nerves. It is a simple, inexpensive, and safe treatment method that does not cause addiction. High-rate conventional TENS has a short duration of action and can be used daily for long periods. Its analgesic effect is produced through gating at the spinal cord. Other settings of TENS, including low-rate (acupuncture-like) TENS and burst-mode TENS, provide analgesic effects for a longer duration. The analgesic effects of these settings are attributed to the release of endorphins. TENS is commonly used as an analgesic agent in both chronic knee and spinal problems, and is tolerated well by patients.^[4,5]^

The stress hormones include adrenocorticotropic hormone (ACTH), cortisol (COR), prolactin (PRL), growth hormone (GH), and catecholamines, most of which are insulin (INS) antagonists.^[6,7]^ The blood levels of stress hormones increase under stressful conditions such as trauma, surgical intervention, and infection. However, the release of stress hormones can also be influenced by exposure to heat and the application of electrical current. As a superficial heating agent, a sauna bath heats all areas of the body, and therefore can cause thermal stress. In the literature, there are also limited studies concerning the effects of electrical current on the endocrine system. In the current study, we examined the hormonal effects of the combined application of hot packs, US, and TENS to the lumbar region and knees in patients with OA. This combination therapy is commonly used both in medical rehabilitation and in pain management.

## 2 Materials and methods

The study was approved by the institutional ethical committee. Forty-four volunteer inpatients with symptomatic grade II or III knee OA according to the Kellgren–Lawrence radiographic grading system and lumbar degenerative disorder were enrolled in this study from January to November 2009 for the evaluation of changes in glucose, INS, GH, PRL, ACTH, and COR levels during 10 sessions of physical therapy at the Rehabilitation Clinic of Abant Izzet Baysal University. All patients provided informed consent. The exclusion criteria were testosterone or postmenopausal estrogen treatment, current neurological rehabilitation, history of surgery or major trauma in relevant regions, presence of inflammatory rheumatic disease, joint inflammation or effusion, suspicion of infection or increased C-reactive protein and erythrocyte sedimentation rate, cognitive dysfunction, malignant tumor, diabetes mellitus, hypothalamus and pituitary gland diseases, adrenocortical disorders, insulinoma, symptomatic coronary arterial disease, myocardial dysfunction, cardiac arrhythmias, cardiac pacemaker use, venous or arterial thrombosis, uncontrolled hypertension, history of syncope, or symptomatic chronic pulmonary disease. All female patients were postmenopausal.

### 2.1 Physical therapy application

Each patient underwent a total of 10 sessions of combination physical therapy including TENS, US, and hot pack. The sessions were carried out once a day, 5 days per week, at the hospital. All sessions were performed by the same competent physical therapist. High-rate TENS was applied by using a 4-channel transcutaneous electrical stimulator device (Elletronica Pagani, Paderno Dugnano, Italy), with a pulse frequency of 100 to 150 pps and a pulse duration of 50 to 80 μs. Self-adhesive percutaneous electrodes (5 × 5 cm) were placed over the area where pain was most intense on palpation. Continuous US (Elletronica Pagani) was applied to all patients at 1 MHz frequency and 1.0 W/cm^2^ intensity by using an applicator with a 5 cm diameter. After the patient was placed in the correct position, an acoustic gel with no pharmacologically active material was spread over the entire application area. US was performed with a longer duration on anatomical sites with the most pain (mostly the medial tibiofemoral compartment of the knee and depending on the levels of the spinous process showing tenderness), with a circular motion of the probe at appropriate angles. Typically, each session included 20 minutes of hot-pack application to the knees and lumbar region, 20 minutes of TENS application to both knees and the lumbar region, and 5 minutes of continuous US application to both knees and to both paraspinal lumbar regions. Thus, in each session, a total of 60 minutes of TENS, and 20 minutes of continuous US was applied to 4 different body areas. The duration of each session was approximately 90 minutes. All patients received isometric quadriceps and pelvic tilt exercises throughout the therapy period.

### 2.2 Venous sampling

To examine the acute effects of physical agents during the 1st session, a 15 mL venous blood sample was withdrawn from the antecubital vein in 44 patients both immediately before the session at 10.00 am, with at least 2 hours of fasting, and following a 5-minutes resting period after the end of the therapy session (group I). A subset of these patients (group II) supplied 15 mL fasting venous blood samples at 8 am before the 1st session (day 1), after the 5th session (day 6), and after the 10th session (day 11).

### 2.3 Laboratory assessment

The blood samples were centrifuged at 1300 *g* for 10 minutes, and the plasma sample for ACTH measurement was immediately separated and analyzed. The serum samples were immediately frozen and stored at −80 °C (1–11 months) until needed for further analysis. All samples were analyzed at the same accredited laboratory by using a chemiluminescence assay (Immulite 2000; Siemens, Glyn Rhonwy, Llanberis, Gwynedd, UK). The homeostasis model assessment index of insulin resistance (HOMA-IR) was calculated with the following formula: serum glucose (mg/dL) × serum INS (μIU/mL)/405. A calculated value of >3 may indicate INS resistance.

### 2.4 Patient assessment

The patients were evaluated for pain and functionality before the 1st therapy session (1st day), and on the 6th and 11th days of therapy. The visual analogue scale (VAS) for pain, Roland Morris Disability Questionnaire (RMDQ) for low back pain, and Western Ontario and McMaster Universities Arthritis Index (WOMAC) for knee pain were used for the evaluation.

#### 2.4.1 VAS for pain

Low back pain and pain in the knees were evaluated separately with VAS during range of flexion motion while the joint was actively flexed by the patient along its entire range of motion. The VAS is a linear scale ranging from 0 to 100 mm, with the left pole (0) corresponding to no pain and the right pole (100) corresponding to unbearably intense pain. The patients were asked to indicate their level of pain by adding a mark on the scale.

#### 2.4.2 RMDQ

The RMDQ is used to examine the functional status of the lumbar spine in the presence of low back pain.^[8]^ It is a subjective scale consisting of 24 self-reported questions on patient status that are easy to answer within a relatively short period. The patients mark the statements that correspond to their physical states. Any statement that does not match their physical state is left unmarked. Every marked statement is scored as 1 point. The total score varies between 0 (no incapacity) and 24 (maximum incapacity). The validity and reliability of the Turkish version of the RMDQ have been confirmed.^[9]^

#### 2.4.3 WOMAC index

The WOMAC index is a subjective scale for examining pain and loss of physical functions due to OA.^[10]^ It is aimed at evaluating chronic problems of the knee and hip joints. The index consists of 3 separate parts. It includes a total of 24 questions that are easy to answer within a reasonably short period. Five questions are aimed at pain symptoms, 2 questions are aimed at joint stiffness, and 17 questions are aimed at physical function. There are 5 selections in the Likert scale version of WOMAC that the patient can mark (0 = none, 1 = mild, 2 = moderate, 3 = severe, and 4 = extreme). After the patient completes the test, each subsection is summed separately, and the total sum is calculated and taken into account. Higher scores indicate poorer health and physical function. The validity and reliability of the Turkish version of the WOMAC index have been confirmed.^[11]^

### 2.5 Statistical analyses

Statistical analysis was performed with MedCalc software, version 13.0.2 (Acacialaan 22, Ostend, Belgium). Descriptive data are expressed as means ± standard deviations. Nonparametric variables were determined by means of the Kolmogorov–Smirnov test. The evaluation of 2-related variables was performed with the paired samples *t* test for parametric variables and with Wilcoxon signed-rank test for nonparametric variables. Analysis of repeated measurements was performed with the repeated measures *t* test for parametric data. When a difference was detected between groups, a Bonferroni adjustment for multiple comparison tests was used to determine from which group the difference originated. Friedman test was used for nonparametric data, and when a difference was detected between groups, Wilcoxon test was used to determine from which group the difference originated. Differences of hormonal changes in units and changes of therapy monitoring indicators in units between sexes on the days of assessment were evaluated by using Student *t* test for parametric variables and the Mann–Whitney *U* test for nonparametric variables. Pearson and Spearman correlation tests were used to analyze the linear relationship between hormonal changes and changes of treatment-monitoring indicators in units throughout the therapy period. Values of *P* < 0.05 were accepted as statistically significant for all parameters.

## 3 Results

This study included patients aged between 58 and 73 years who had a diagnosis of symptomatic knee OA and degenerative lumbar spinal disorder. Most study subjects were characterized with a high body mass index (BMI) and low vitamin D status. The hormonal effects of physical agents before and after the 1st therapy session were determined in 44 patients including 10 (22.7%) men and 34 women (group I). The mean age of the patients in group I was 64.9 ± 4.8 years, and their mean BMI was 30.7 ± 3.5 kg/m^2^. After the application of physical agents, there was a 23.2% decrease in INS levels and a mild but statistically significant decrease in PRL levels (*P* = 0.001 and *P* = 0.041, respectively). There was an 80.4% increase in GH levels, but this increase was not significant (Table 1). Both the increase in GH levels and decrease in PRL levels were greater in men than in women; however, only the difference in the decrease in PRL levels was statistically significant (*P* = 0.044).

**Table 1:**
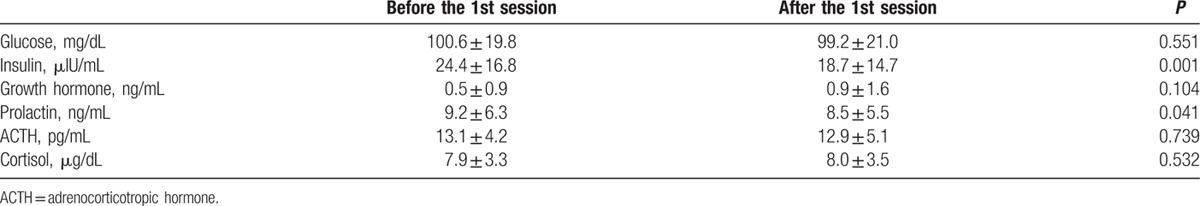
Changes in biochemical variables caused by the initial therapy in group I patients (n:44).

Thirty-seven patients agreed to continue with the 2nd part of the study (group II). These patients provided serum samples once every 5 physical therapy sessions (in a total of 10 sessions). There were 10 (27.0%) men and 27 women in group II, whose mean age was 64.3 ± 4.7 years and mean BMI was 30.5 ± 3.4 kg/m^2^. At the end of the therapy period, there was a 27.0% improvement in the VAS-spine scores, an 18.9% improvement in the RMDQ scores, a 34.5% improvement in the VAS-knee scores, and a 24.8% and 29.1% improvement in the WOMAC-total and WOMAC-pain indices of the patients, respectively. The INS levels increased by 31.8% throughout the therapy, and the ACTH and COR levels decreased by 24.1% and 21.0%, respectively, reaching their trough levels. The changes in the hormonal and treatment-monitoring indicators throughout the therapy period are shown in Table 2. There were no statistically significant differences between the sexes in terms of changes in hormonal and treatment indicators; however, the elevation of PRL was more pronounced in women than in men, and the 11th session INS and HOMA-IR values were also slightly higher in women (*P* > 0.05 for all). Table 3 shows the linear relationships between the changes in the units of ACTH and COR levels and changes in the units of treatment-monitoring indicators between the 1st and the 11th day, which was the following day of the last session. There was a positive correlation between changes in both hormones and changes in the all treatment-monitoring indicators. However, only the changes in COR levels and WOMAC-pain score were statistically significant (*P* < 0.05). No complications were observed throughout the therapy period.

**Table 2:**
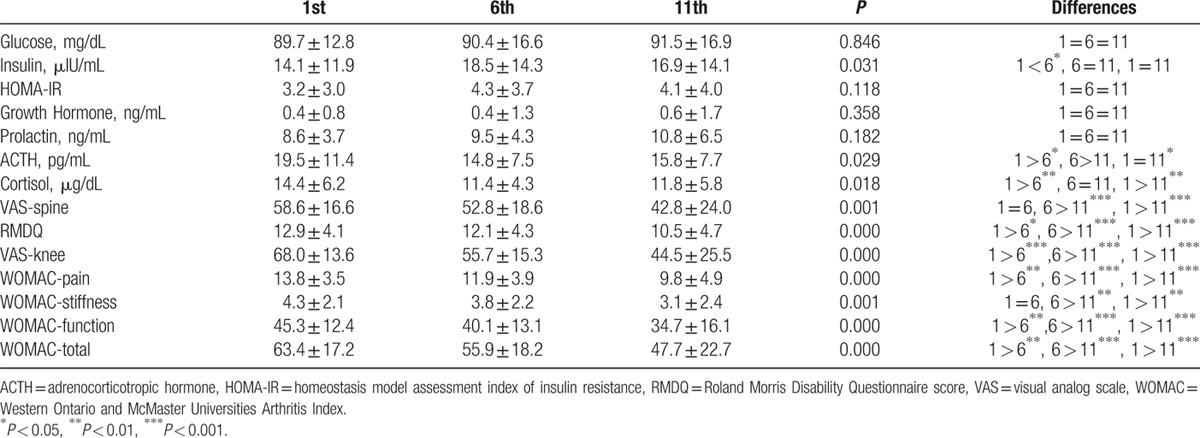
1st, 6th, and 11th day fasting levels of biochemical variables and values of treatment monitoring indicators in group II patients (n:37).

**Table 3:**
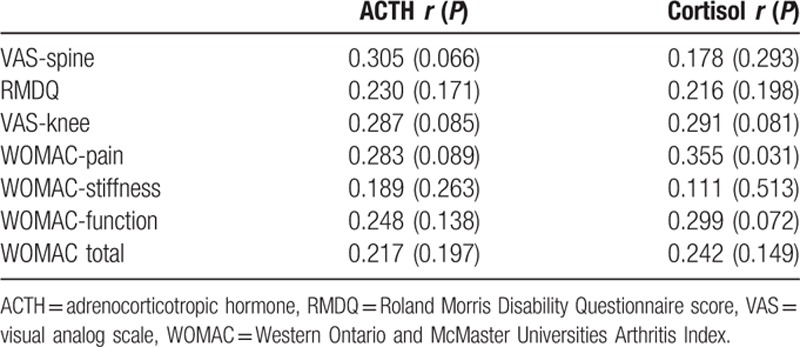
Correlation of the changes in ACTH and cortisol levels with changes in treatment monitoring indicators in group II patients between the 1st day and 11th day (n:37).

## 4 Discussion

We observed that the combination therapy reduced the complaints related to the knee and lumbar region. There was a delay in the decreases in the VAS-spine and RMDQ scores compared with the decreases in the VAS-knee and WOMAC scores. This may have been due to insufficient penetration because the lumbar structures are located more deeply than the knee joint. In group I patients, we observed that the INS levels decreased by approximately 23.2% after the 1st session, but the serum glucose levels did not change. The INS levels increased up to 31.8% at the 6th day of the therapy in group II patients who were also mostly overweight or obese; however, increase in INS resistance as represented by HOMA-IR had not developed. Both the levels of ACTH and COR decreased permanently by >20%. The decrease in these 2 hormones can be attributed to the analgesic effect of the physical therapy agents. Furthermore, this may be an indicator of patient comfort caused by a central antistress effect or the development of an adaptive response.

The effects of physical therapy agents on the endocrine system have not been fully elucidated. Published data have not sufficiently clarified the effects of electrotherapy modalities, in particular TENS, on the endocrine system. It has been reported that TENS application does not cause a significant change in ACTH and COR levels in healthy volunteers.^[12]^ However, serum COR levels were reported to decrease by 40.8% during electric acupuncture at nonacupuncture points in patients with chronic low back pain.^[13]^ However, to our knowledge, there are no published reports relevant to local heating agents.

There are studies reporting the effects of a sauna bath on the endocrine system. Sauna baths involve the whole body and have a superficial heating effect. The studies reported a 2- to 5-fold increase in PRL levels, a 2-fold increase in GH levels, a 30% to 65% increase in ACTH levels, and insignificant changes in COR levels that varied according to sex in healthy adults immediately after using a sauna bath at 80 °C for 20 minutes. The glucose and INS concentrations were not affected by the sauna bath.^[14,15]^ Nonetheless, in another study, researchers performed an oral glucose tolerance test during the sauna bath, and the results indicated a decreased glucose utilization and INS response.^[16]^ This result was attributed to decreased visceral circulation, sympathetic activation, and elevated concentrations of anterior hypophyseal hormones during the sauna bath. In another study, a hot water bath was applied to healthy male volunteers at 39.5 °C for 2 hours, and the results indicated a 16-fold increase in GH, a 67% increase in glucagon, and a 10% increase in glucose levels, whereas no change was detected in INS levels.^[17]^ Koska et al^[18]^ found that head-out water immersion at 38 to 39 °C for 25 minutes resulted in a 13-fold increase in GH and a nearly 50% increase in PRL levels. Leppaluoto et al^[19]^ found that a 2-hour dry sauna at 80 °C for 7 days caused moderate decreases in serum COR and plasma ACTH levels in healthy males. In that study, there was a 16-fold increase in GH and up to a 2.3-fold increase in PRL, whereas the GH levels decreased after the 3rd day. These studies indicate that sauna and hot-water baths cause rapid and marked changes in endocrine system functions. Fluid electrolyte loss is also considered to contribute to the development of hormonal changes.

Heat is a strong stimulus for PRL and GH secretion. The serum levels of these hormones increased many fold during hyperthermia in both sexes, although differences in individual response may be present. Indeed, GH may be the hormone most sensitive to heat stimulus. GH stimulates sweat production and evaporation during heat exposure. GH-deficient adults have a decreased ability to normalize their body heat by evaporation during exposure to thermal stress, which is one of the reasons for their decreased exercise capacity.^[20]^ GH replacement therapy normalizes sweat secretion and increases fluid and sodium reabsorption in the renal tubules. Acute administration of GH results in decreased urinary electrolyte and fluid excretion. Chronic GH administration has more diverse effects, which involve a combination of primary and secondary effects of GH and their compensatory mechanisms.^[21]^

Aldayel et al^[22]^ observed an increase in the serum GH levels, reaching a peak of 4-fold at 15 minutes of an electrical muscle stimulation that can induce 40 isometric muscle contractions with pulsed current (75 Hz, 400 seconds) or alternating current (2.5 kHz delivered at 75 Hz, 400 seconds); however, they did not observe a significant change in the serum COR levels. Another study reported that the GH response to exercise-induced heat is higher in highly heat-tolerant athletes, whereas the increases in COR and norepinephrine in these subjects are more moderate.^[23]^ Therefore, in this current study, the 80.4% increase in GH after the initial session, which was not statistically significant, may be attributed to the instant effect of the heating stimulus of US and hot-pack application for some patients.

Significantly increased PRL levels indicate the development of fatigue induced by heat exposure, and thermal stress is known to cause increased PRL levels.^[24]^ This condition is thought to be related to central noradrenergic activation. Therefore, the moderate decrease in serum PRL levels after the 1st session in group I suggests that combination therapy does not cause stress in the patients. Moreover, analgesia occurring during TENS application may have caused temporary decreases in PRL levels, especially in men. PRL secretion may be influenced by prompt changes in the current pain status as an acute response. It has been reported that high PRL levels are associated with psychological stress and musculoskeletal pain.^[25,26]^ Patients in group II had nonsignificant increases in PRL levels throughout the 10-session therapy period, which might be due to increases in INS levels. Because high circulating levels of PRL are associated with impaired glucose regulation, it has been suggested that PRL is protective against diabetic complications.^[27]^

Persons with diabetes may have greater susceptibility to adverse effects from heat.^[28]^ However, there are limited data in the literature concerning the effects of heat exposure on carbohydrate metabolism. Heat exposure decreases basal INS and glucose concentrations in sheep, partly through β-adrenergic modulation.^[29]^ It has been reported that INS injection directly stimulates warm-sensitive neurons in the preoptic area of the hypothalamus in a dose-dependent manner, causing a thermogenic effect.^[30]^ Thereby, the observed decrease in INS levels after the 1st therapy session may be attributed to heat changes induced by US and hot-pack applications, although it is difficult to detect rapid changes in INS levels due to the moderately decreasing trend of this hormone level in the periods before meals, concordant with serum glucose levels. The serum glucose level did not distinctly decrease after the therapy session. Conversely, recurrent heat exposure may have caused mild INS resistance in the following days, particularly in susceptible patients. Therefore, in group II patients, thermal stimuli may have caused the increased INS levels. Other factors contributing to increased INS levels may be the antinatriuretic effect of this hormone and the decreased serum COR levels. Corticosteroids are responsible for salt retention through renal sodium reabsorption, and COR also decreases sodium loss through the small intestine in mammals.^[31]^ Therefore, the INS levels may have increased in order to compensate for the disturbances in sodium balance caused by evaporation and the decreased COR levels. However, it has been suggested that the antinatriuretic effect of INS only appears in cases of hyperglycemia or INS resistance.^[32]^ INS resistance was evident in the patients of this study.

This study has several limitations. First, this study did not include a control group. Moreover, it was impossible to apply the hot packs in a placebo setting, and there is no appropriate placebo for TENS. Patients can typically feel the electrical current under the electrodes. As OA is a chronic disorder, many patients had been previously treated with physical agents and may share their therapeutic experiences with each other. Second, the results could have been better reviewed if the levels of catecholamine, glucagon, thyroid hormones, and B-endorphin could be measured. Additionally, the different endocrine effects of therapeutic modalities could have been demonstrated separately if TENS or only a heating agent were applied as monotherapy rather than as combination therapy. Nonetheless, we observed that this combination physical therapy, widely used in clinical practice and applied to different regions with short-duration US therapy, may be used safely in terms of hormonal changes, particularly in nondiabetic patients.

## 5 Conclusions

We demonstrated that the studied physical agents are well tolerated by patients with OA, and their therapeutic effects may be reflected in pituitary-adrenal axis function, which can be considered an indirect evidence of efficacy. The combination therapy seemed more efficient for knee pain than for low back pain when applied to each region in the same period. This therapy program failed to trigger hormonal alterations that demonstrate stress status, both in the 1st therapy session and throughout the therapy period. Conversely, the hormonal responses may have been blunted owing to the age of the patients. In addition to changes in INS secretion, the combination treatment with these agents did not adversely affect the endocrine functions of patients. Changes in INS secretion were not reflected in serum glucose levels. Nevertheless, the therapy should be applied with caution in diabetic patients. Further studies are needed to clarify the effects of physical therapeutic agents on the endocrine system. The hormonal effects of other therapeutic agents used in clinical practice should additionally be examined.

## Acknowledgments

The authors thank Mrs Sengul Gunduz for her technical assistance to laboratory work.
